# Alike but also different: a spatiotemporal analysis of the older populations in Zhejiang and Jilin provinces, China

**DOI:** 10.1186/s12889-023-16433-w

**Published:** 2023-08-11

**Authors:** Lei Jiang, Xingyu Chen, Wenjie Liang, Bo Zhang

**Affiliations:** 1 School of Geography and Remote Sensing, Guangzhou University, Guangzhou, 510006 China; 2 Guangdong Provincial Center for Urban and Migration Studies, Guangzhou, 510006 China; 3 School of Economics, Zhejiang University of Finance and Economics, Hangzhou, 310018 China; 4 Southern Marine Science and Engineering Guangdong Laboratory (Zhuhai), Zhuhai, 519000 China

**Keywords:** Population ageing, Older population, Spatial distribution, Geographical detector, Socioeconomic factors, Comparison analysis

## Abstract

According to the 7^th^ National Population Census, China is experiencing rapid growth of its ageing population, with large spatial disparities in the distribution of older folks in different regions. And yet, scant comparative research has been conducted on the two regions of Zhejiang and Jilin in particular, which differ considerably in economic development but witness nearly the same ageing trend. In response, this article compares Zhejiang, an advanced economic province, with Jilin, with its relatively low level of economic development, to explore the ageing issue and analyse the spatial correlation between older populations and socioeconomic factors. Using the spatiotemporal data analysis and geographical detector approaches, we obtain three significant findings: 1. both provinces have maintained steady rates of increase in ageing; 2. the older populations in Zhejiang and Jilin are mostly concentrated in the provincial capitals and nearby cities with reasonably established economies; and 3. the factors, including local fiscal expenditures, beds in hospitals and nursing homes, and coverage of social security, show a highly similar spatial pattern between older populations in Zhejiang and Jilin. The *q*-values of all the selected socioeconomic factors in Jilin showed a growth trend, indicating that the spatial correlation between these factors and ageing is strengthening year on year, that is, the resources gained from the socioeconomic development of Jilin have shifted steadily toward old-age services. As a consequence, a vicious circle of the slowing down of the economic growth drives away working forces and quickens the pace of population ageing, is present. From a policy perspective, Jilin province is strongly dependent on state-owned enterprises characterised by institutional rigidity, an inflexible market economy and an under-developed private sector, all of which are profoundly influenced by ageing. The consequence is large population outflows of young people. In contrast, the economy of Zhejiang province is partially decoupled from the ageing trend, so the gap in level of development between its counties has been narrowing. The policy implication here is that Zhejiang represents an active private economy that has coped successfully with ageing by attracting young migrants and developing new forms of development, such as the digital economy.

## Introduction

China’s ageing population has been accelerating since 2000. The number of older people over age 60 has increased from 10.5% in 2000, to 18.9% in 2021, and this rising trend is expected to exceed 30.0% by 2035 [1]. The old-age dependency ratio also shows a meteoric rise in the elderly population, from 9.9% in 2000 to 19.7% in 2021, and is projected to reach 49.9% in 2049, nearly 6% higher than in OECD countries [2]. The Chinese ageing trend is predicted to be even faster than Japan within the next 25 years [3]. Moreover, with improvements in living standards and medical care, the average life expectancy of Chinese people rose from 71.4 years in 2000 to 78.2 years in 2021 [4]. However, as the number of older people continues an upward trajectory, care provision for the aged is increasingly becoming a serious social issue. Furthermore, the one-child policy and changing views on raising children and perpetuating the family lineage are also being reshaped [5]. Indeed, it is noteworthy that the total fertility rate (TFR) has dropped below the replacement of 2.1 in 1992 (considered as the critical degree to sustain the population growth) to 1.3 in 2020. These demographic trends are expected to lead to sharp declines in the labour force and greater demand for public services and support for the elderly.

Despite China’s entry into a demographic stage marked by ageing, there are large spatial differences across provinces. According to the NBS [4], the ageing issue in North China is more serious compared to South China. A likely reason is that the one-child policy over the past 30 years was carried out more strictly in the north [6]. A general demographic change since 2000 indicates that internal migration in China takes place from north to south and from inland to coastal cities, resulting in an unbalanced population distribution because most migrants are relatively young [7]. Statistics from the 7^th^ Census also show that the three northeastern provinces have the highest percentage of residents age 60 and older, with 25.7% in Liaoning, 23.2% in Heilongjiang and 23.1% in Jilin, respectively. In the eastern part of China, 23.4% of the population in Shanghai and 19.6% in Beijing are over age 60.

However, the ageing situation is considerably less severe in the western region, with the exception of Sichuan and Chongqing; in year 2000, in the population group of age 65 and over, these two provinces ranked seventh and tenth in the country, respectively. According to the 7^th^ Census of 2020, they are now ranked second and third after Liaoning, but in Qinghai, Xinjiang and Tibet, ageing populations represent 12.1%, 11.3%, and 8.5%, respectively. The pace of ageing varies greatly across different regions in China, rising most notably from west to east, and strongly associates with regional economic prosperity, advanced urbanisation and large-scale migration [8]. The wide variation in the Chinese ageing population indeed yields significant socioeconomic and spatiotemporal consequences and challenges.

Therefore, mapping the spatiotemporal distribution of population groups is vital to our understanding of how the population is aggregated or dispersed within a specific period. In particular, it would be useful to identify variations in ageing populations, note changes in different geographical spaces, and visualise the process onto maps reflecting ‘ageing hotspots’ in different regions at different stages [9]. Rishworth and Elliott [10] also emphasise the importance of geographical factors and their interrelation with spatiotemporal characteristics of the ageing process. Correspondingly, other authors argue that the spatial distribution and the agglomeration of the ageing population is generally sensitive to geographical scales because, for instance, when the ageing population is agglomerated and calculated at provincial level, it is likely that the ageing individuals among the local population will be overlooked [11].

Socioeconomic development is arguably the main reason contributing to the spatiotemporal heterogeneity of the ageing population [12–17]. In a global scale survey test for spatiotemporal heterogeneity, Wan et al. [18] mapped a geospatial aggregation of older people. Socioeconomic factors comprised 61.85% of the survey’s explanatory power. Furthermore, the ageing groups are usually disadvantaged and suffer more due to their marginalised identities and diminished socioeconomic circumstances [19]. A study of the relationship between socioeconomic factors and the ageing process, and an evaluation of how these factors influence the spatiotemporal distribution of the ageing population would thus garner important information.

At the global level, Wang [20] finds in a study from 1990 to 2010, the distribution of world ageing populations presents as high-high clusters in most of the European and North American countries and Russia, but low-low clusters in many African countries through analyses of global and local spatial autocorrelations by using a spatial data analysis approach. In particular, the urbanisation rate and life expectancy is likely to lead to the growth of the ageing population in the countries and their neighbouring countries. For policy makers, high-high and low-low clusters indicate that policy makers should consider regional-based ageing care policies rather than state-based ones among adjacent countries. In addition to socioeconomic factors having a persistent and positive impact on the distribution of the aged, Wan et al. [18] incorporate environmental factors in their study, they confirm that climate change, green space, national income, air quality, and sex ratio are critical explanatory elements for the geospatial distribution of ageing population at global scale. Jarzebski et al. [21] combine population ageing and shrinking together, arguing that context-specific socioeconomic interventions should be performed to minimise the risk of ageing issues and to meet the UN’s sustainable development goals (SDGs). In other words, although population ageing is a global issue, measures should be taken at local level in accordance with unique geographical conditions.

At national level, Man et al. [22] analyse how socioeconomic factors have influenced the movement of gravity centres of the ageing population in China between 2002 and 2018. Main findings show that gravity centres of the elderly population rate (EPR) and elderly dependency ratio (EDR) have moved to the northeast, but urbanisation trends and economically relevant factors have shifted to the southwest, showing an opposite trend to the ageing population [22]. Other research by Feng et al. [23] points to urbanisation, not population density, which is enlarging the regional gaps in China in terms of ageing populations. Furthermore, in many other developed countries, the growth rate of ageing populations in peripheral areas is usually higher than in the urban centres [24]. The spatial distribution of the ageing population in China is similar to a developed country such as Japan at the same urbanisation level [25]. However, Zhang et al. [5] argue that although socioeconomic factors, including per capita GDP and per capita disposable income are positively related to the spatial distribution of the ageing population at national level, these same factors can be negatively related at provincial level. Whereas at regional level in the Chinese Yangtze River Delta region, Xu et al. [17] identified that migration, per capital GDP and road network density are the most influential elements contributing to the spatial heterogeneity of population ageing.

Ageing trends are reported to be exaggerated when socioeconomic and environmental factors interact. In a study of four Chinese mega cities, Beijing, Shanghai, Guangzhou, and Wuhan, Xie et al. [26] found a bidirectional concentration tendency of the ageing population at city scale. One shows centrifugal spread where seniors move from city centres to suburbs. The other is centripetal concentration where seniors move from suburbs to city centres. This two-way movement is actuated by the ‘selective development’ model of local government: upgrade of urban infrastructure and income discrepancies among older people’s pensions [25]. In reality, the impact of socioeconomic factors on older populations across China is complicated. As discussed above, research on population ageing can be roughly divided into two categories: analyses of the older population and the degree and characteristics of ageing from a spatiotemporal-geographical perspective to verify the unbalanced spatial distribution of the ageing population, and studies on the quantitative relationship between the ageing and the socioeconomic factors at macro level through different statistical modeling approaches with policy recommendations.

However, scant comparative research has been done on two regions with starkly different levels of economic growth, but which nevertheless suffer from a similar ageing issue. Therefore, this article compares Zhejiang, one of the most economically developed provinces in China–to Jilin province, which has a relatively low level of economic development–in an analysis of the ageing issue at county scale. We address the following questions: 1. What is the current state of the ageing population in the Zhejiang and Jilin provinces, and how is it distributed over space and time? 2. Is there a correlation between socioeconomic factors and population ageing in Zhejiang and Jilin? We first derive data from the county level then conduct a variety of spatial data analyses to ascertain the distribution of older populations in both provinces. We also identify the two provinces that will be affected by the demographic ageing crisis. Next, we apply a spatial stratified heterogeneity (SSH) analysis method using the geographical detector model to analyse how socioeconomic factors and increased ageing populations are distributed spatially in Zhejiang and Jilin. Thereafter, we discuss differences if any. In our comparison of the two provinces, we aim to shed light on the causes of the disparities between them, and in so doing, allow for the development of plausible policy proposals that might be implemented elsewhere in China.

## Locating Jilin and Zhejiang province in the age of ageing: Different economic development stages but similar ageing conditions

**Fig. 1:**
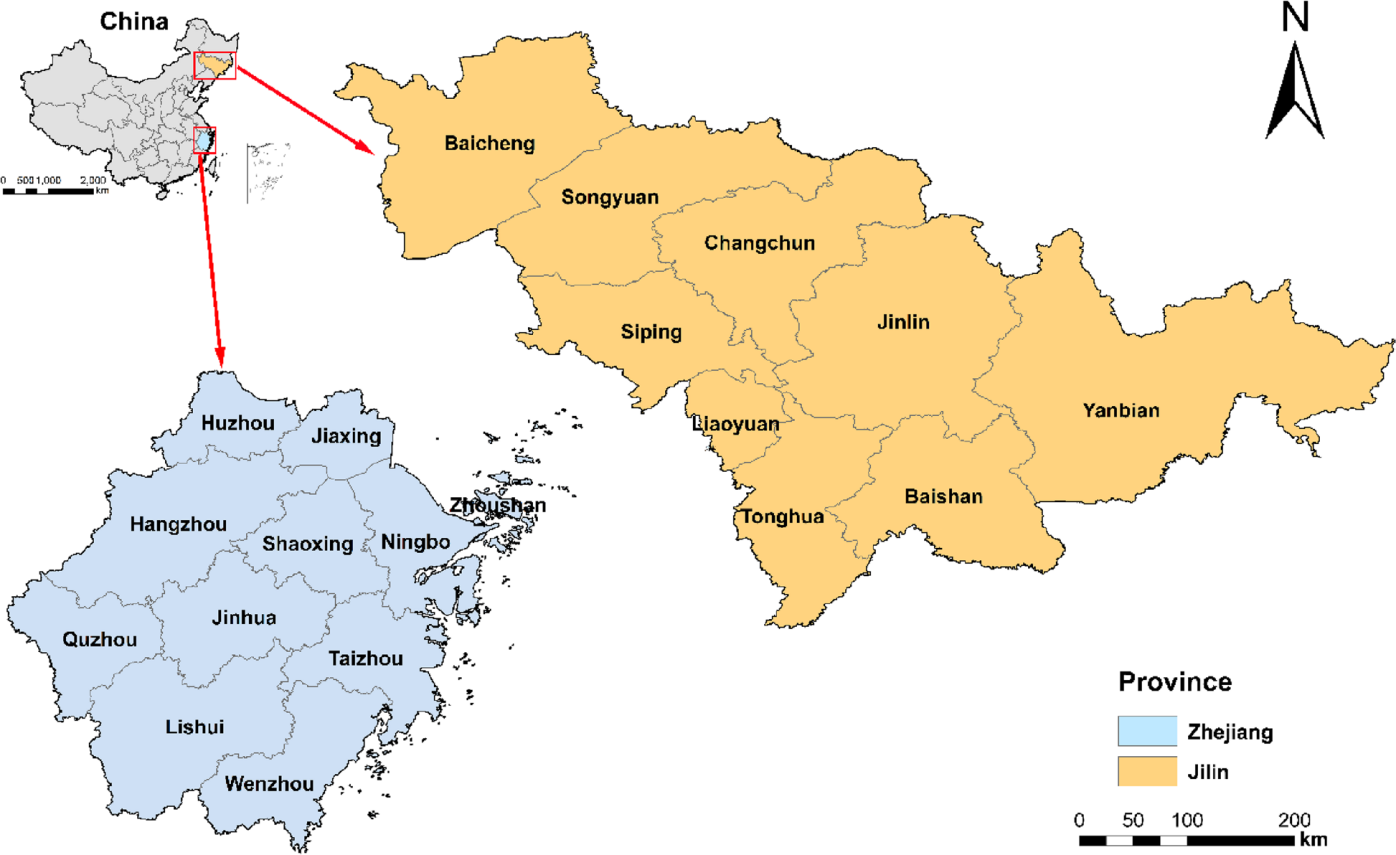
Demo of study area: locations of Jilin and Zhejiang provinces in China

Despite the large ageing populations in both provinces, Zhejiang has sustained a rapid economic growth, whereas Jilin has remained economically stagnant. To find the underlying causes for this phenomenon we apply a comparative analysis to explore how regions with different levels of economic development respond to the problem of population ageing. Zhejiang is at the forefront of reform and opening of the Yangtze River Delta (Fig. 1); it has a vibrant private enterprise economy and, in recent years, has the third-highest per capita income behind Beijing and Shanghai. Its robust economic and social growth has not only set a standard for other provinces but has also resulted in a range of improvements, including increased urbanisation rate, higher level of educational attainment, gradual change in care concepts for children and older adults, and a remarkable rise in life expectancy. These factors have enabled Zhejiang to lead the way in addressing the ageing population problem among other provinces.

Jilin, a relatively underdeveloped industrial province in the northeast (Fig. 1), is also facing a serious ageing issue as its economy declines and population shrinks. Since the launch of reform and opening up, the conventional paradigm of economic development in the northeastern region, which was historically dependent on heavy industry and agriculture, has gradually collapsed. Furthermore, new economic forms such as the digital economy and the internet economy have developed much more slowly in Jilin than in the southeast region, thus contributing to chronic economic failure. Importantly, Jilin has also seen the lowest birth rate in the country due to its strict enforcement of family planning policies; this situation exacerbates the decline of the working-age population and propels a higher ageing rate. Over three decades the pace of population growth in Jilin has slowed steadily and lagged behind the national average. What’s more, cities like Daqing and Anshan, which are heavily reliant on natural resources, even saw a negative growth rate in the population [27]. In addition, the mass exodus of young people from the less developed cities in Jilin province, who left in search of employment with higher wages in more economically developed provinces, has accelerated the problem of the ageing population [28].

**Fig. 2:**
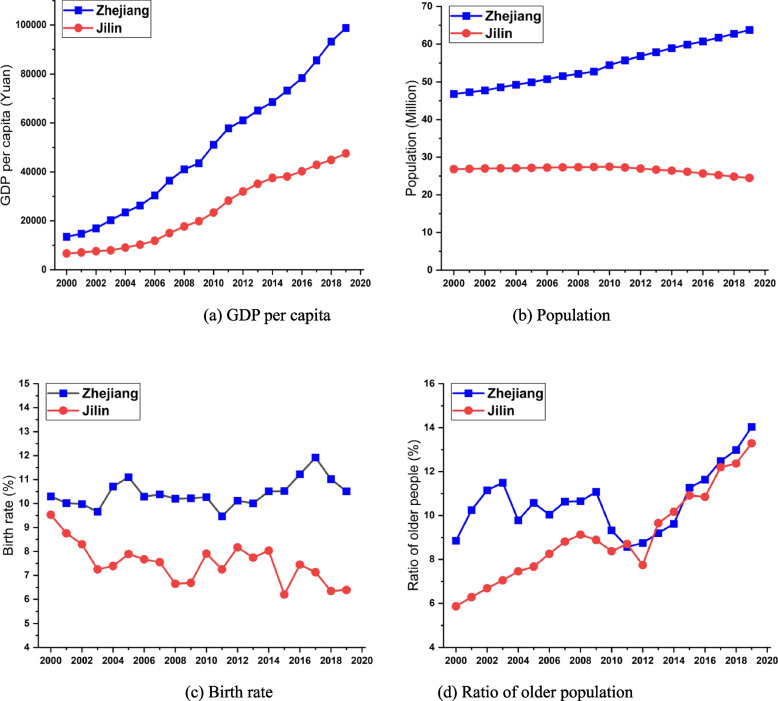
Comparison of basic information on demographic and economic indicators between Zhejiang and Jilin provinces

Figure 2 depicts the changes in the basic socioeconomic characteristics of Zhejiang and Jilin provinces from 2000 to 2020. When comparing per capita GDP, Zhejiang is slightly higher than Jilin in 2000, but is approximately 2.5 times more prosperous than Jilin in 2020. In terms of gross population, we observe a mild increase in Zhejiang and a gentle decline in Jilin. Compared to Jilin, the birth rate in Zhejiang was only a little higher in 2000, but the divergence is wide in 2020. Interestingly, despite the discontinuation of the national government’s one-child policy in 2016, we can observe that the birth rate of Jilin still drops in 2017. The implication here is that Jilin has not benefited from preferential policy in terms of its population growth. The ratio of the older population to the total is higher in Zhejiang than Jilin in 2000, but by 2012, indicates the ratio of the older population in both provinces has been increasing with little difference in 2020. From Fig. 2, we can see that although both Jilin and Zhejiang have experienced substantial ageing trends during the previous decade, their economic performances have diverged dramatically.

## Research methods and data description

Next, we investigate the spatial distribution patterns and characteristics of older populations in Zhejiang and Jilin at county level to identify which areas are witnessing the greatest rates of population ageing. Thereafter, we apply the geographical detector approach to assess the degree of similarity between the number of older people and socioeconomic factors in terms of spatial distribution. Following this, we conduct an analysis of the respective economies in these two provinces to explore whether such a demographic issue of an ageing population could pose a challenge to the expansion of the economic landscape. Lastly, we again employ the geographical detector approach to examine the extent to which the distribution of ageing population is explained by the selected socioeconomic factors through the lens of spatial stratified heterogeneity.

### Geographical detector analysis

Geographical detector analysis, first proposed by Wang et al. [29], can determine the correlation between two variables from a perspective of spatial stratified heterogeneity. When there is a significant correlation between two variables, the degree of spatial similarity between them will be greater. The geographical detector method has seen recent widespread application to geo-referenced data in many fields, such as urban landscape structure [30], children’s welfare homes [31], health expenditure [32], tuberculosis [33], tourism demand for destinations [27], hospitalised acute lower respiratory infections [34], sustainable livelihood security [35], and rural economic restructuring [36].

In the geographical detector method, factor detection is used to describe the extent to which the explanatory variable *X* affects dependent variable *Y*, that is to say, it measures the influence of *X* on *Y*, which is denoted by the *q*-statistic, as follows

1 $$q=1-\frac{{\Sigma }_{h=1 }^{L}{N}_{h}{\sigma }_{h}^{2}}{N{\sigma }^{2}}=1- \frac{SSW}{SST}$$

2 $$\mathrm{SSW}={\Sigma }_{\mathrm{h}=1}^{L}{N}_{h}{\sigma }_{h}^{2}$$

3 $$\mathrm{SST}=N{\upsigma }^{2}$$

where *h* represents the stratum of variable *X* or *Y*; *N*_*h*_ and *N* are the number of spatial units for stratum *h* and the study area, respectively; *σ*_*h*_^*2*^ and *σ*^*2*^ represent the sample variances for stratum *h* and *y*-value of the study area, respectively; and *SSW* denotes the sum of the variances associated with the stratum, while *SST* denotes the sum of the variance of the whole sample. *q* takes a value between 0 and 1. An increase in the *q*-value represents a larger impact of variable *X* on *Y*, giving greater explanatory power. In the most extreme scenario, when *q* = 0, variable *X* is independent of *Y*, and when *q* = 1, variable *Y* is entirely dependent on *X*.

The geographical detector approach, featured by the perspective of spatial stratified heterogeneity, is suitable for our research due to the following two reasons. One is that it can identify and measure the spatial stratified heterogeneity of the distribution of ageing population. As shown in Figs. 4 and 5, the ageing population of the two provinces are not evenly distributed, indicating remarkable spatial disparity. The other is that it can easily quantify the association between ageing population and socioeconomic indicators when it posits that spatial stratified heterogeneity tends to be consistent without the linearity assumption [29].

### Data description

In this study the ratio of people age 60 and older is derived from the statistical yearbooks of Zhejiang and Jilin as an indicator of population ageing. In accordance with the relevant literature discussed in the introduction and data availability, six primary socioeconomic factors are selected to analyse the spatiotemporal similarities and correlations between economy, infrastructure and number of older people, namely, local fiscal expenditures (*Fiscal*); beds in health institutions (*Hospital*); beds in nursing homes (*Nursing*); per capita GDP (*PCGDP*); number of persons covered by social security (*Security*); and gross provincial product of Zhejiang and Jilin provinces (*GDP*). Our selection of these factors is guided primarily by the present state of economic growth and the availability of infrastructure to meet the basic needs of older people. However, due to the unavailability of statistical data on the coverage of social security in Jilin, the corresponding fiscal expenditure was used instead.

The number of older populations, as well as socioeconomic variables for 73 counties in Zhejiang, and 47 counties in Jilin between 2004 and 2018, was derived from the statistical yearbooks of Zhejiang and Jilin provinces. However, due to the data availability, explanatory variables in the county level in both provinces are not available in the districts in urban areas. For instance, the explanatory variables in the counties in Hangzhou are available but in the districts in urban areas (Shangcheng district, Xiacheng district, Gongshu district and etc.) are not available. Therefore, the data from urban areas (Shangcheng district, Xiacheng district, Gongshu district and etc.) is summed together in the yearbooks. Multiple statistical datasets therefore had to be merged into a single set. Specifically, the number of older adults in urban districts of each city at prefecture level was combined into an urban sample. This was also how we handled the socioeconomic variables. In short, county level datasets are used for the spatiotemporal analysis of older populations, while aggregated data from urban districts and counties are used in the geographical detector model. However, data unavailability only allows us to apply the geographical detector model with data from 2014 to 2018.

## Spatiotemporal variation of older populations in Zhejiang and Jilin provinces

### Overall ageing trends in Zhejiang and Jilin provinces

**Fig. 3:**
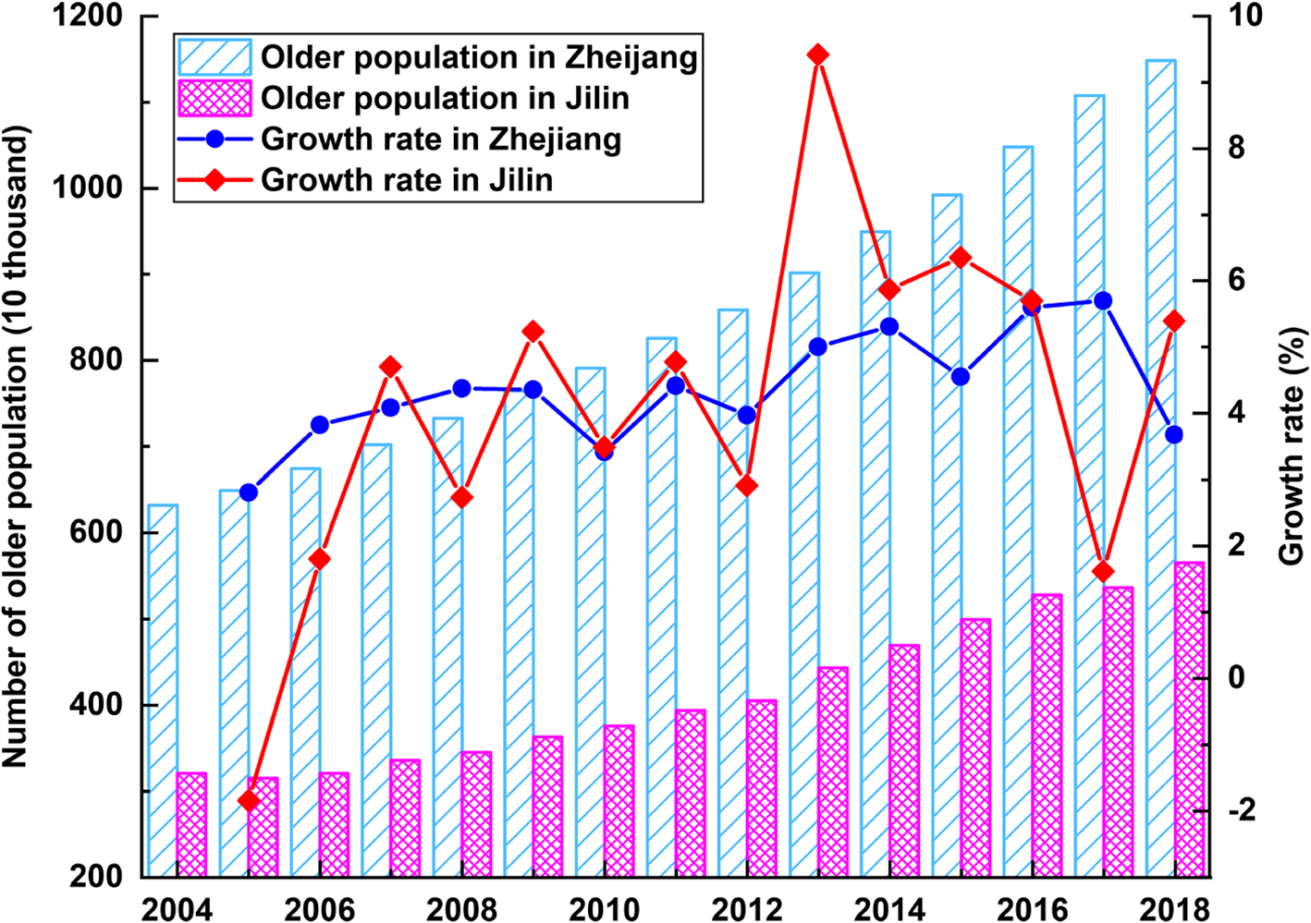
Number of older populations and average annual growth rates in Zhejiang and Jilin provinces

We first created a chart that visualises the total number of older populations and their annual growth rates in the provinces of Zhejiang and Jilin between 2004 and 2018, as shown in Fig. 3.

We can observe that, from 2004 to 2018, the population growth rate in Jilin fluctuated dramatically, whereas it climbed with slight fluctuations in Zhejiang. Overall, the ageing population in both provinces has maintained an almost linear growth pattern. The growth rate of Jilin, however, is significantly higher than Zhejiang when we compare the growth curves. Specifically, the growth rates of the two provinces were positive, suggesting a continual rise in the number of older persons in both provinces, except for 2005, the only year indicating a negative growth rate of Jilin’s older population. Between 2005 and 2018 we observe a 4.36% annual increase in the older population in Zhejiang: with a peak of 9.42% in 2013 and a trough of -1.84% in 2005. Jilin’s yearly growth rate nevertheless averaged 4.12%. Whereas Zhejiang has a larger older population than Jilin, approximately three times higher than Jilin between 2004 and 2010. Thus, Zhejiang will have to cope with a greater challenge to its provision of eldercare.

### Spatiotemporal variations of older populations in Jilin and Zhejiang provinces

**Fig. 4:**
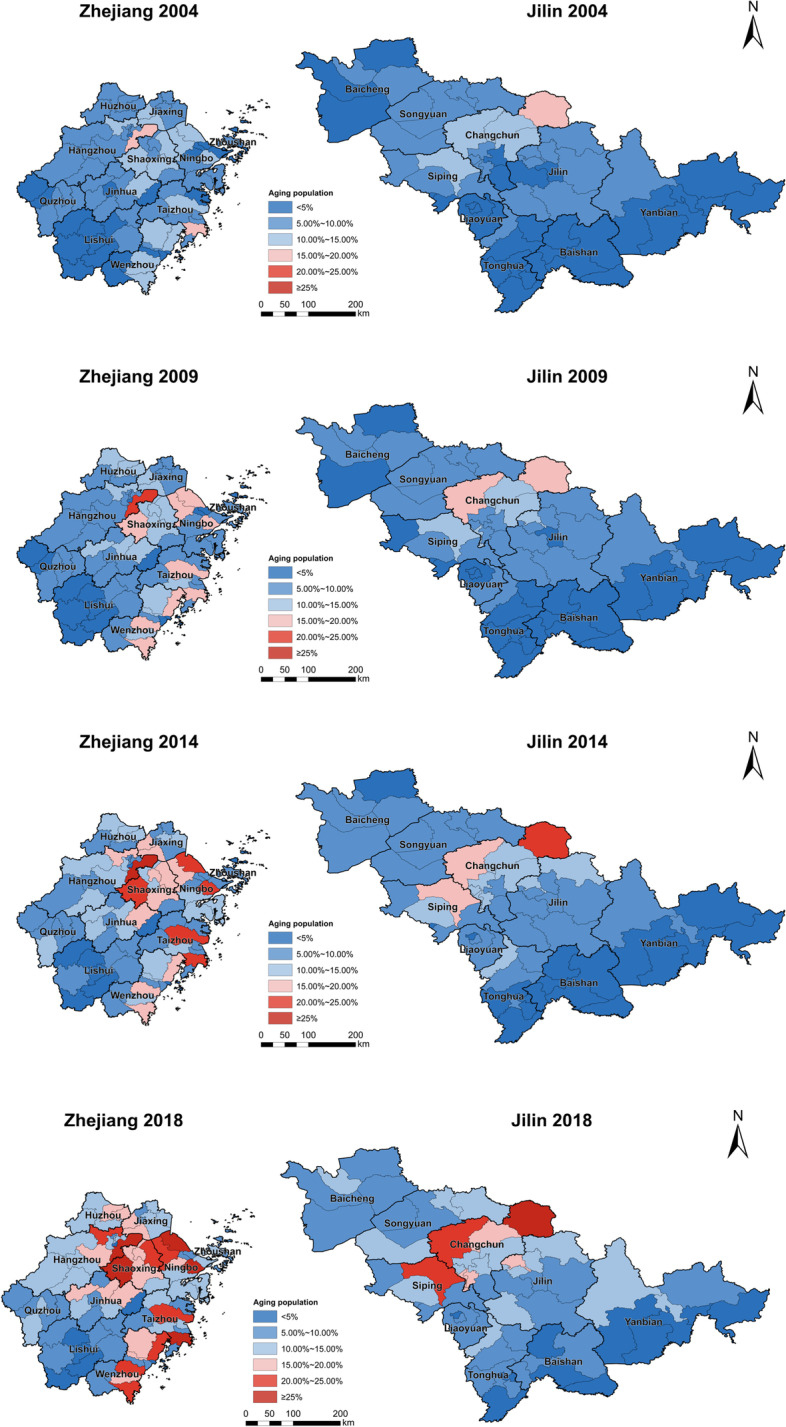
Spatial distribution of the ageing population by county in 2004, 2009, 2014, and 2018: Zhejiang and Jilin

**Fig. 5:**
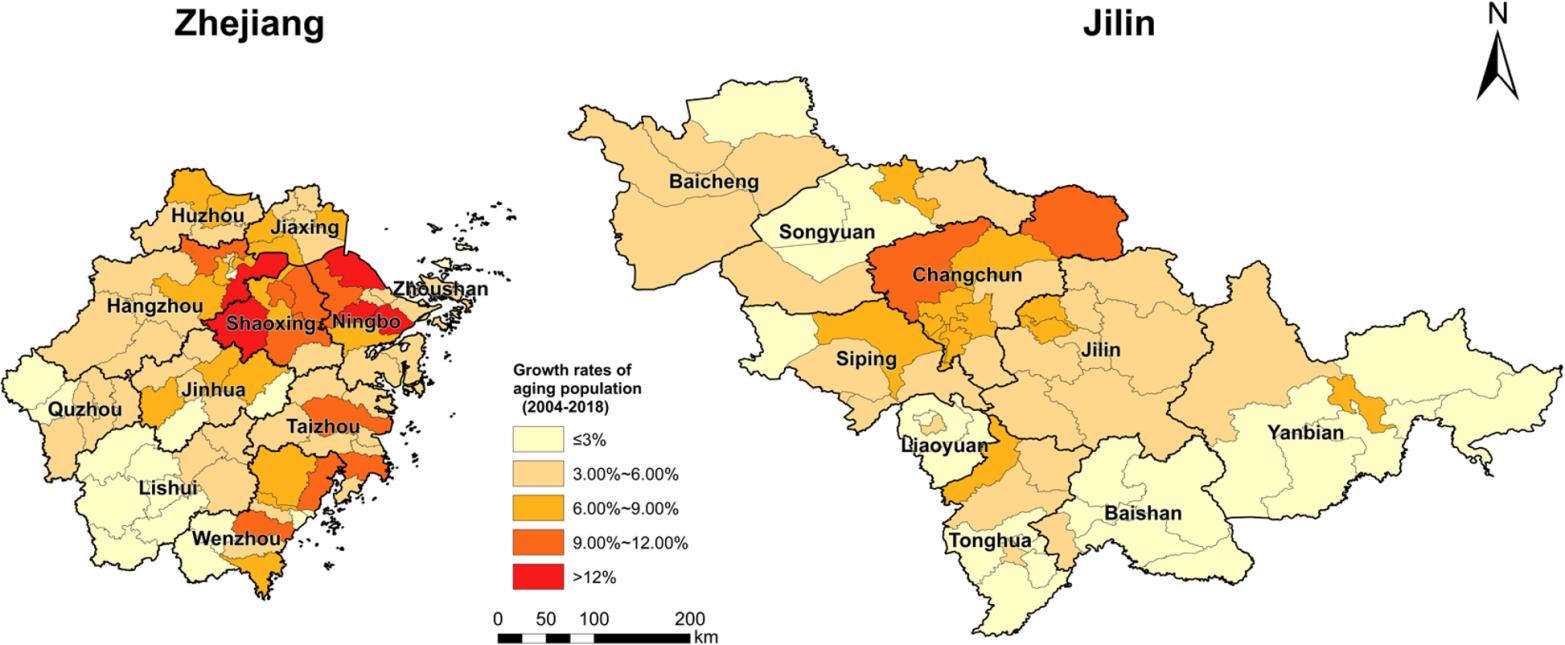
The growth ratio of ageing populations between 2004 and 2018: Zhejiang and Jilin

We present below the spatial distribution (Fig. 4) and the growth rate (Fig. 5) of older people at county level in Jilin and Zhejiang provinces between 2004 and 2018.

When we turn to the spatial distribution of the ageing population in Zhejiang, in 2004 only two counties have an ageing population higher than 15%. By 2018, 30 counties^1^ have higher than 15% ageing populations. Generally, in Zhejiang, the economically developed Hang-Jia-Hu Plain, the Ning-Shao Plain and the Wenzhou-Taizhou Region have the densest distribution and the highest growth rates of seniors from 2004 to 2018. According to the spatiotemporal variations presented in Figs. 4 and 5, it is reasonable to conclude that economic and social development and older population are similarly distributed in space; namely, they have a strong spatial similarity and are therefore strongly correlated.

In Jilin, the spatial distribution of the ageing population is aggregated in the capital city Changchun and the surrounding area. In 2004, only one county, Yushu, has an ageing population higher than 15%, but by 2018 the number of the counties where seniors comprise up to 15%, increases to eight^2^. Counties with the highest growth rate of the older population are located in the capital city and the growth rate declines from Changchun to its surrounding areas. What’s interesting here is that, in Jilin, there is only one concentrated centre of elderly in the capital city Changchun. However, in Zhejiang, there are three concentrated centres of older people, including the socioeconomically highly developed Hang-Jia-Hu Plain, Ning-Shao Plain and Wenzhou-Taizhou region. In sum, the distribution of older people in two provinces shows similar trend, namely, older people are spatiotemporally concentrated to the socioeconomic more developed areas. It is then safe to say that the socioeconomic development and the spatial aggregation of older people are highly and positively correlated.

## Results of the geographical detector analysis

### Results of the Pearson correlation coefficients

As a first step toward conducting the geographical detector analysis, we compute the Pearson correlation coefficients as a benchmark method between older populations and socioeconomic variables for Zhejiang and Jilin provinces, respectively, to determine whether they are linearly correlated. Results are given in Table 1.

**Tab 1:** Results of Pearson correlation coefficients

Year	Province	*Fiscal*	*Nursing*	*Hospital*	*Security*	*PCGDP*	*GDP*
2014	Jilin	0.964	0.947	0.959	0.872	0.320	0.917
	Zhejiang	0.745	0.750	0.785	0.761	0.573	0.792
2015	Jilin	0.941	0.367	0.938	0.896	0.286	0.905
	Zhejiang	0.742	0.704	0.783	0.776	0.571	0.793
2016	Jilin	0.942	0.940	0.944	0.894	0.331	0.908
	Zhejiang	0.789	0.774	0.778	0.801	0.434	0.815
2017	Jilin	0.963	0.960	0.950	0.913	0.494	0.924
	Zhejiang	0.789	0.868	0.767	0.788	0.509	0.806
2018	Jilin	0.971	0.966	0.966	0.888	0.562	0.945
	Zhejiang	0.797	0.850	0.765	0.795	0.489	0.813

All results were significant at the 1% significance level or lower

As can be seen in Table 1, except for the variable of *PCGDP*, the Pearson correlation coefficients of Jilin between the other five independent variables and the dependent variable are relatively high. Most coefficients are greater than 0.9. In other words, these socioeconomic factors have a robust linear relationship with population ageing in Jilin province. Similarly, the Pearson correlation coefficients between the six socioeconomic variables and the dependent variable in Zhejiang are slightly lower, mostly between 0.70 and 0.87, which likewise suggests a strong linear association. Thus, from the above analysis, we can conclude that the variables *Fiscal*, *Nursing*, *Hospital*, *Security*, *PCGDP*, and *GDP*, are all positively and linearly correlated with the number of older populations in both Zhejiang and Jilin.

### Results of the geographical detector analysis

**Fig. 6:**
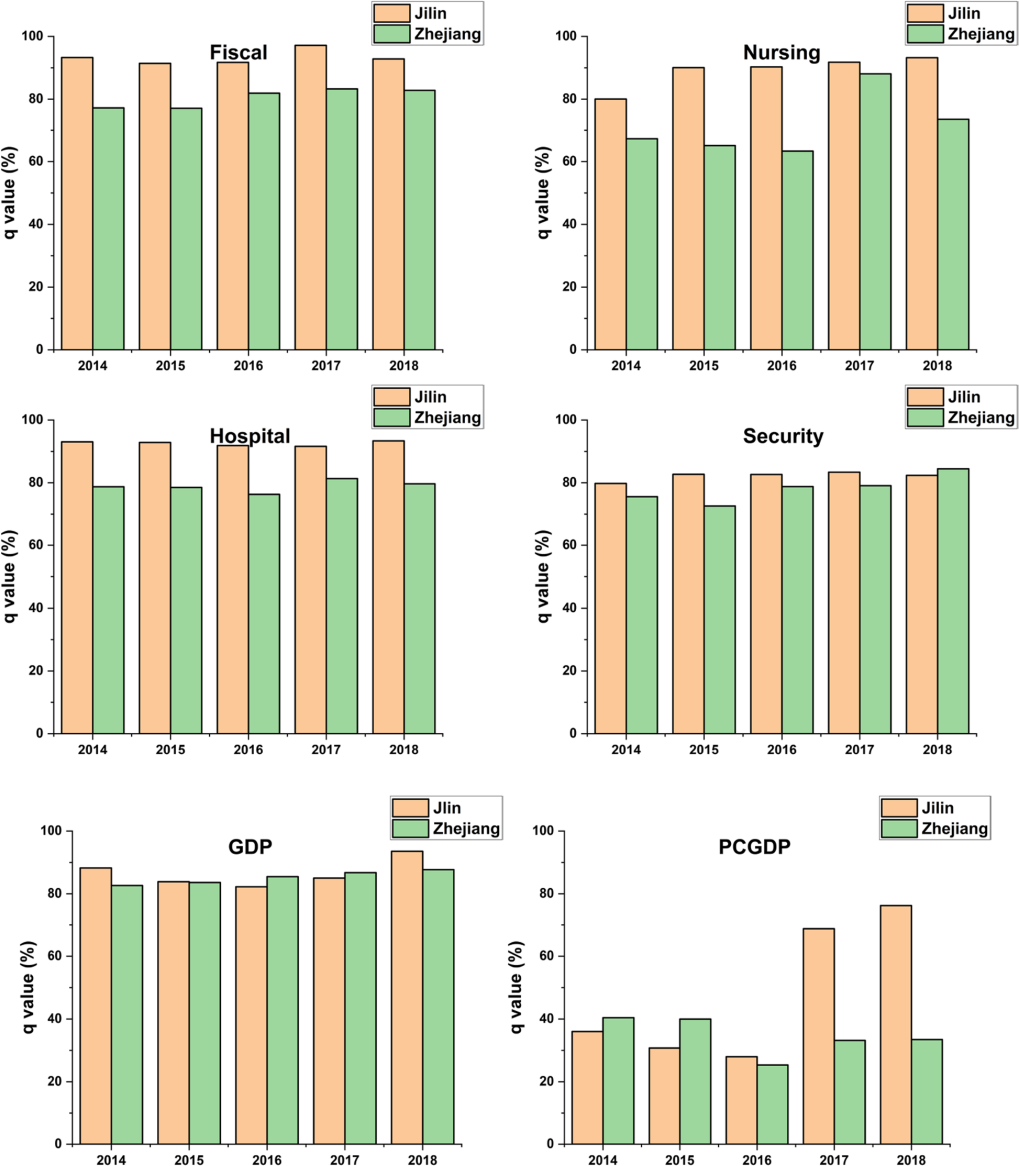
Results of factor detector analysis. Note: All *q*-values are statistically significant at the 1% level

The Pearson correlation coefficient, as a measure of the strength of the linear association between two variables, has been widely applied in research. However, it may suffer from a shortcoming in the present study; it does not consider spatial stratified heterogeneity. To overcome this shortcoming, we use the geographical detector to assess any similarities between the spatial distribution of the ageing population and socioeconomic factors in the two provinces. Results of the analysis are presented in Fig. 6.

In Fig. 6, most factor scores (i.e., *q* values) of variables of Zhejiang fall in the 0.63 to 0.88 range, with *PCGDP* being the outlier; whereas all variables of Jilin score far above 0.9, with the highest *q*-value reaching 0.971 (e.g., *Fiscal* variable of 2017), indicating that number of older people in Jilin is more strongly correlated with socioeconomic factors in terms of spatial similarity. Meanwhile, such exceptionally high scores reflect that the ageing population in Jilin has been adequately served by economic infrastructure and social security, thus revealing a coordinated relationship between the ageing population and public services. Conversely, the high scores also suggest that this rapidly graying demographic poses a serious challenge to socioeconomic progress. In other words, socioeconomic development in Jilin is tightly constrained by the ageing issue.

Judging from the *q* factor scores over the years, all six independent variables of Jilin exhibit a general upward trend. One can infer that the spatial correlation between socioeconomic factors and population ageing has been steadily intensifying over time. In particular, factor scores related to social welfare and security, such as *Fiscal* and *Nursing*, have risen significantly. However, the variables *Hospital*, *PCGDP* and *GDP* show a mild U-shaped pattern in the direction of the changing trend. Further, there is minimal variation in the factor scores of *Hospital* and *Security*, showing a fairly steady spatial correlation between the two. On the contrary, growth in Zhejiang is only visible in the analysis of three independent variables, namely *Fiscal*, Security and *GDP*, while the variables *Nursing* and *Hospital* appear to fluctuate. The *PCGDP* variable even shows a downward trend. In general, Zhejiang has lower *q* factor scores than Jilin across the board for the six variables; the implication here is that Zhejiang can cope more effectively with an ageing population. Meanwhile, Jilin has gradually shifted its social resources toward care and support services for older people due to higher numbers of seniors. More socioeconomic resources are therefore needed to keep up with demographic changes in Jilin.

### Discussion of differences in economies between Zhejiang and Jilin provinces in the context of ageing

**Fig. 7:**
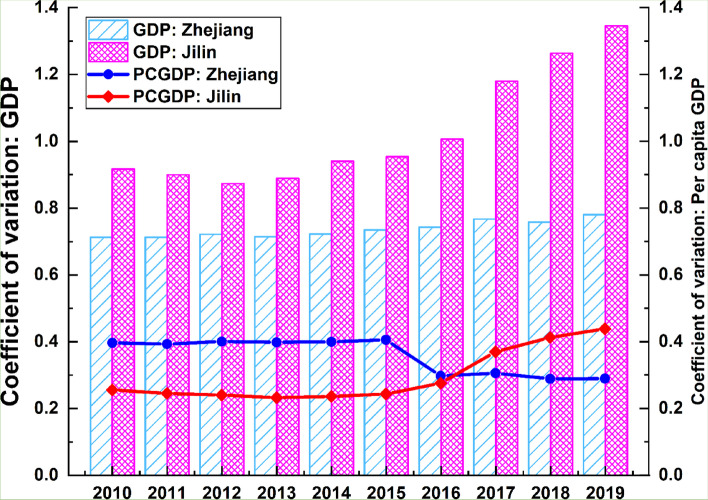
CVs of GDP and PCGDP of Zhejiang and Jilin provinces from 2010 to 2019

Of the six socioeconomic factors discussed above, all are strongly associated with the ageing population except for *GDP* and *PCGDP*. Since the majority of older people are concentrated in the economically developed capitals of the two provinces and their surrounding regions, the *GDP* variable exhibits a significant degree of spatial similarity with the older population. The *PCGDP*, however, is quite modest. In view of this, we apply the coefficient of variation (CV), defined as the ratio of the standard deviation to the mean, to analyse the extent of the variability of *GDP* and *PCGDP* for Zhejiang and Jilin provinces. Thereafter, we discuss in-depth the context of the combination of the economic development models and industrial structures of the two provinces. Results are presented in Fig. 7 below.

From 2012 to 2019, the CVs of *GDP* of Jilin shows a continuously increasing trend, suggesting that the economic gap across cities has widened over time. However, the CVs of *GDP* of Zhejiang grow slightly before 2017, then shrink and virtually stay constant afterward, indicating that differences in *GDP* across cities in Zhejiang have remained relatively steady in recent years. In the case of *PCGDP*, the CVs of Jilin climb rapidly between 2016 and 2019, while in Zhejiang there is slow growth before starting decline in 2015. Zhejiang and Jilin display entirely distinct growth patterns regardless of any comparison between the CVs of *GDP* or *PCGDP*. In sum, spatial agglomeration and polarisation of economic growth in Jilin are a result of the expanding disparities in total GDP and the trajectory of economic development between the various regions of the province. In contrast, the CVs of *GDP* of Zhejiang reveal a steady rise, indicating that GDPs in different parts of the province remain in close step with one another.

Figures 4 and 5 illustrate how Jilin’s economic growth model and industrial structure are directly connected to the striking similarities between the spatial distribution of both GDP and the older population. At the outset of the country’s founding, Jilin was a pioneer in the development of resource-intensive heavy industry and the establishment of numerous state-owned enterprises (SOEs). The local economy relied heavily on these SOEs, and as a result, socioeconomic resources tended to be concentrated in the SOE areas. While the market economy has shown its vitality with deeper reforms and greater opening up, some large SOEs have been unable to restructure to accommodate market conditions. As natural resources have gradually been depleted, resource-based cities like Liaoyuan have seen their economic development receive less support and attract fewer human and material resources than during the planned economy era; as a result, the coordination between population structure and economic development has been gradually declining [28]. As the 1990s era of market economies emerged, the slow decline of cities with a single industrial structure and state dependency became apparent. The economies of some large cities, including the provincial capital of Changchun, fell markedly. Surrounding cities were impacted too,their limited access to resources resulted in increased migration away from these cities. The growing older population, coupled with the outmigration of young people in recent years, has contributed to the high spatial similarity between the economically developed cities and population ageing. According to the development trend of the factor scores and the CVs of the *PCGDP* variable, the income gap in Jilin will continue to widen. However, economically developed cities are also experiencing greater strains on the care provision for older residents.

The situation in Zhejiang tells a different story. Decreasing CVs indicate that the difference in per capita GDP of Zhejiang is gradually becoming smaller; so is the gap in living standards across regions. Since the reform and opening up policy, Zhejiang’s model of economic growth has been distinctly different from Jilin’s. Due to its lack of natural resources and arable land, Zhejiang has become the most active region for private sector activity in China, and the most impressive performances have come from its coastal cities, namely, Hangzhou, Shaoxing and Ningbo. Moreover, the economically prosperous parts of Zhejiang province have helped to accelerate the development of other relatively economically backward regions, resulting in a parallel growth pattern in recent years. The economic development of Zhejiang tends to be balanced and all-encompassing. Since the population density in the plains of northern Zhejiang is much higher than in the mountainous area of southern Zhejiang, the more developed the economy becomes, the larger becomes the older population in spatial terms. Conversely, the recent widespread development has led to a narrowing of the income gap across regions in Zhejiang; thus, the *q*-values of the *PCGDP* variable and the older population are lower. Also, as the income level of less economically developed areas in southern Zhejiang increases, the degree of spatial similarity between the two will tend to decrease.

The comparison of Jilin and Zhejiang reveals that, despite a more severe ageing issue in economically developed areas, the development trends of the two provincial capitals diverge significantly. The robust private sector in Zhejiang has largely contributed to its economic vitality, as seen by total economic output and per capita GDP, both far higher than Jilin. Moreover, the strong economic development momentum of Zhejiang has attracted many young people from outside the province, thus optimising the age structure of the population. The larger influx of young labourers has made Zhejiang’s economy more dynamic, and the prosperous economy is better situated financially to support its ageing population. In contrast, the region of Northeast China (Jilin), dubbed the ‘eldest son of the Republic,’ which has long relied on heavy industry and agriculture to develop its economy, has failed to make a smooth transition to a market economy. At the same time, rather than fostering rapid economic growth, the concentration of socioeconomic resources in relatively developed and economically dense regions has worsened the division between them, thus stifling economic growth in Jilin. The dual effects of a lagging economy and an ageing population further compound the problem. Due to the growing issue of graying demographics, a greater proportion of financial and social resources will have to be allocated to aged care, which has tended to crowd out market investments. As the population ages, fewer young people will be available to work, which in turn exacerbates the economic lag. The steady decline in employment and opportunities in Jilin, and the consequent massive migration of young people to other developed provinces have further contributed to the deterioration of the economy and the ageing of the population. All in all, the combination of economic stagnation and population ageing in Jilin has led to a higher share of societal resources being devoted to the elderly care sector and fewer financial inputs to support productive industries.

To better understand the nexus between ageing and economic growth, we have also introduced a degree of decoupling (DD) approach to measure de-linking economic growth from increasing ageing issues [37]. DD has already been applied in energy and carbon emissions research. Specifically, in this research it is defined as the percentage change of ageing population divided by the percentage change of GDP from 2000 to 2019. It can be expressed as

4 $$DD=\%\Delta Aging/\%\Delta GDP$$

Results show that the *DD* value of Jilin is 0.2367, twice that of Zhejiang (0.1170). This finding confirms that Zhejiang’s economy is less constrained by the ageing issue than Jilin, given that they have similar ageing trends, most notably in the recent decade from 2010 to 2019 (See Fig. 2d above).

### Differences in social security expenditure between Zhejiang and Jilin provinces in the ageing context

**Fig. 8:**
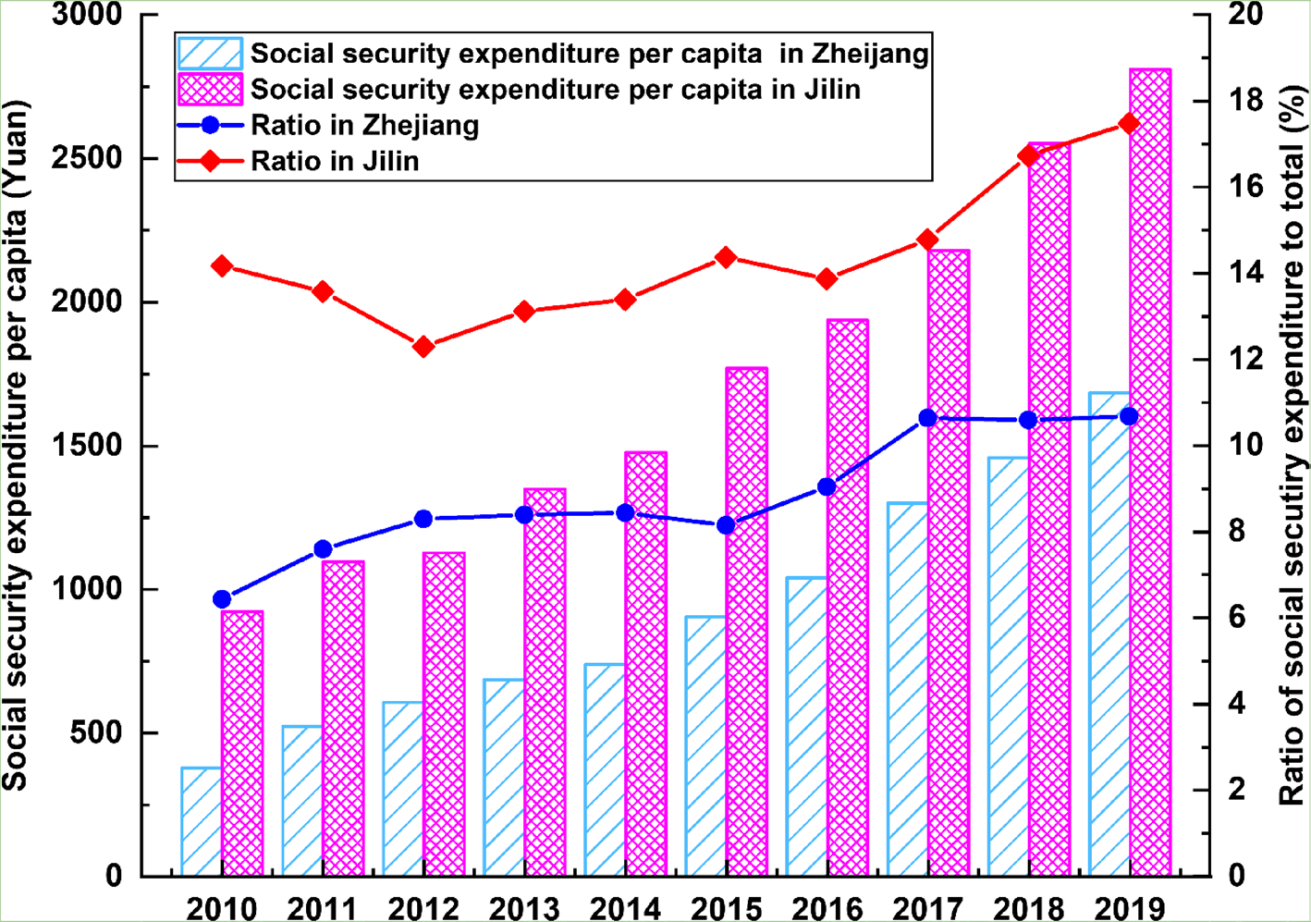
Proportion of fiscal expenditure for social security to provincial GDP and GDP growth rates of Zhejiang and Jilin provinces from 2010 to 2019

In a further analysis, the proportion of social security expenditure and the rate of change in economic growth over time for the two provinces are shown in Fig. 8 below.

In Jilin, the percentage of social security spending to total fiscal expenditures is substantially greater than that of Zhejiang, suggesting that Jilin has to dedicate significantly more financial resources to the aged care sector. In terms of temporal patterns, the percentage of social security expenditure in Zhejiang underwent a period of expansion from 2015 to 2017, followed by a modest fall in 2018. On the whole, it was in good shape, but future declines might occur. In Jilin the story is different; spending on social security comprised a significant share of its total fiscal expenditures and it was also growing rapidly. Interestingly, despite showing signs of improvement in 2016, the economic growth of Jilin plummeted to unprecedented lows. Hence, this gives credence to the argument made earlier, that the economy of Jilin is stagnating. The decrease in the young labour force and the rise in the older population, have each contributed to the fast-graying demographics in the northeast region of China, leading to a profoundly negative impact on economic growth. As far as public fiscal expenditures are concerned, the percentage to GDP in the northeast region has dropped year on year since the 1980s, and notably, this trend has accelerated in the past 10 years [4]. The slow growth of its economy will hinder Jilin’s ability to manage the ageing issue on its own, and the province is highly likely to depend increasingly on financial transfers from the central government.

In contrast, Zhejiang has maintained a strong economic growth rate of over 6% between 2010 and 2019, and the trend remains upward. Comparatively speaking, due to the significant gap in economic development between the Jiangsu-Zhejiang-Shanghai area and the northeastern region (including the provinces Liaoning, Jilin and Heilongjiang), young and exceptional talent from the northeastern provinces have sought employment in the developed regions. Research verifies that regions with advanced economies, higher salaries and better medical and educational conditions have succeeded better than others in attracting migrants [38]. Since the reform and opening up policy, the Yangtze River Delta region has seen progressive private sector growth and improved transportation networks, such as railroads, ports and highways, making it one of the most desirable destinations for migrants and a magnet for population aggregation [39, 40]. With its thriving economy and high-quality healthcare facilities and educational opportunities, the Jiangsu-Zhejiang-Shanghai area continues to attract high numbers of talented young labourers. The availability of these superior resources has not only greatly increased economic vitality, but it has also ameliorated the population ageing issue.

## Conclusions and policy implications

The authors have selected Zhejiang and Jilin provinces as their case study to analyse the spatiotemporal variations and socioeconomic factors influencing older populations at county level from 2004 to 2018. Specifically, in the initial step of the present study, the numbers of older people at county levels in the two provinces were shown on maps to determine the changing patterns over time. We then applied spatial stratified heterogeneity (SSH) analysis to measure the degree of spatial similarity between older populations and socioeconomic factors through the geographical detector model. Three main conclusions can be drawn.

(1) From 2004 to 2018, both Zhejiang and Jilin demonstrated an overall trend of expansion in their older populations, with minimal differences in the average growth rates between the two. Zhejiang has a larger older population than Jilin, but both provinces are facing increasingly severe challenges in providing care and support to the aged. (2) In Zhejiang, the counties with high values were mainly located in the north and east, while the counties with low values were clustered in the Lishui mountainous area in the southwest of the province. The cities in northern Zhejiang, such as Ningbo, Hangzhou, Shaoxing, and Jiaxing, had a high annual growth rate for the older population, whereas cities in southern Zhejiang, such as Lishui and Wenzhou, witnessed a low annual growth rate. In general, growth decreases from north to south in geographical terms.

As for Jilin, high values were primarily found in the central region, Changchun and the surrounds, while low values were mostly concentrated near the provincial border in the southeast. In both provinces there was a concentration of older people in the economically developed provincial capitals and their surrounding cities because early on in their development, provincial capital cities were resource-rich with strong industrial economies that enabled them to support higher populations. However, the ageing problem, typified by depopulation at provincial level and over-concentration of population in the regional capital due to a weakened traditional economy and high property prices, has led to large increases in migration outflows of Jilin’s youth. (3) The ageing population in Jilin has become spatially similar over time, and subsequently led to an apparent contraction of economic growth, as indicated by geographical detector analysis. The wider implication here is that, on the one hand, Zhejiang is characterized by a private economy and vibrant market that is coping successfully with the ageing issue through the decoupling of its economic growth from investment in its age-related infrastructure. On the other hand, Jilin has a burdensome historical path dependence on its rigid public sector and heavy industry, which has led to a high correlation between its economic growth and ageing. The result is a vicious cycle of slowing economic growth, which drives away the labour force and quickens the pace of population ageing, ultimately yielding fewer resources for seniors.

Based on the conclusions of the present study, we can suggest several policy recommendations to address the problem of ageing in the two provinces. First, the inflow and outflow of the young labour force is one of the primary causes of the disparity between the provinces of Zhejiang and Jilin. The massive labour outmigration from Jilin, coupled with a low birth rate has resulted in a severe shortage of young workers. Due to the consequences of slow economic growth, lagging fiscal expenditures and an under-resourced medical sector, all essential to the needs of an ageing population, productive activities in Jilin province are tightly constrained. Hence, local policies could emphasise measures to encourage young people to lay down roots. Recent research on internal migration in China finds that access to jobs, secure housing and an abundant supply of social amenities like health care, education, along with higher income, may considerably promote the settlement of the floating population [41]. In response, the government can simplify administration and devolve authority to lower levels, while simultaneously decreasing the conditions for settlement and providing appropriate social resources to attract a younger workforce. Government can also provide employment subsidies for college graduates, housing subsidies and employment of family members. Apart from introducing migrant workers, strict enforcement of retirement policies could reduce the burden on the pension fund. Alternatively, if feasible, the retirement age could also be appropriately raised to supplement the gap between the supply and demand of pensions, given the economic and social conditions. Likewise, talent or start-up subsidies could be used to support the private sector and entrepreneurship to relieve the burden on small and micro businesses. Another possible option is to lure large enterprises to relocate through preferential policies. A measure to alleviate the strain of an ageing population could also be lower taxation and land grants for young talent to in-migrate.

Second, the spatial stratified heterogeneity analysis results reveal that most of the older people in Zhejiang are primarily situated in the Ningbo-Hangzhou area and Wenzhou-Taizhou area, and those in Jilin are mostly clustered in the Changchun-Siping area, both of which are economically developed. So, it is possible to execute pilot programs in these areas to promote the construction of institutionalised services and facilities aiming to reduce costs of eldercare incurred by the government. Specifically, more large public nursing homes need to be built, as well as fees and admission requirements lowered; people living in private nursing homes should also be eligible for subsidies. Moreover, there could be a dominant role for the home-based model in areas with fewer older populations, while centralised care could be built in villages or communities to provide professional services, facilities, and health care to meet the medical and daily needs of older residents. In rural areas, where nursing homes are not readily available, an affordable mutual-aid care system for the aged could be developed using social idle funds and human resources; such an option fits the actual situation and provides a proper solution for rural old-age care. The resolution of these problems may also allow more youth to obtain employment, and in so doing foster economic growth.

Lastly, there is a huge difference between the care provided in urban and rural parts of Zhejiang province from the perspective of the status quo of ageing care. The high concentration of older people in urban areas is likely to put pressure on the facilities such as available beds in nursing homes for the elderly. Whereas the occupancy rate in some remote areas is around 30% [42]. When in need of care, older people in rural areas often have traditional ideas and thus choose at-home care as their first option [43]. In this situation, a financial aid program could be offered to vulnerable groups such as those who depend on subsistence allowances, or have lost their only child, or who receive subsidies and living allowances to encourage them to move into eldercare institutions. Also, a different mode of old-age care is suggested for regions with limited resources. By maximising advantages in urban areas, the government could, for instance, construct more eldercare institutions through financial investment and provide intensive caregiving, as well as expand facilities in the community so that seniors who prefer traditional care can receive home-based care services, thereby allowing them to spend their golden years in the comfort of their own homes.

## Data Availability

The data and material used in this article will be available upon the request from the corresponding author.

## Footnotes

1 Counties include Wuxing and Nanxun county in Huzhou; Tongxiang and Haining county in Jiaxing; Xiaoshan, Yuhang and Fuyang county in Hangzhou; Zhuzhou, Shaoxing, Shangyu, Yuecheng and Chengzhou county in Shaoxing; Yuyao, Cixi, Yinzhou, Haishu and Jiangdong county in Ningbo; Lanxi, Yiwu and Dongyang county in Jinhua; Linhai and Wenling county in Taizhou; and Yongjia, Leqing, Ruian, Pingyang, Longgang, Cangnan, Lucheng and Ouhai county in Wenzhou.

2 Counties include Chaoyang, Nanguan, Erdao, Nongan, Dehui and Yushu county in Changchun; Gongzhuling county in Siping; and Changyi county in Jilin.
